# The Ensembl gene annotation system

**DOI:** 10.1093/database/baw093

**Published:** 2016-06-23

**Authors:** Bronwen L. Aken, Sarah Ayling, Daniel Barrell, Laura Clarke, Valery Curwen, Susan Fairley, Julio Fernandez Banet, Konstantinos Billis, Carlos García Girón, Thibaut Hourlier, Kevin Howe, Andreas Kähäri, Felix Kokocinski, Fergal J. Martin, Daniel N. Murphy, Rishi Nag, Magali Ruffier, Michael Schuster, Y. Amy Tang, Jan-Hinnerk Vogel, Simon White, Amonida Zadissa, Paul Flicek, Stephen M. J. Searle

**Affiliations:** ^1^European Molecular Biology Laboratory, European Bioinformatics Institute, Wellcome Genome Campus, Hinxton, Cambridge CB10 1SD, UK; ^2^Wellcome Trust Sanger Institute, Wellcome Genome Campus, Hinxton, Cambridge CB10 1SA, UK; ^3^Present addresses: The Genome Analysis Centre, Norwich Research Park, Norwich NR4 7UH, UK; ^4^Eagle Genomics Ltd, Babraham Research Campus, Cambridge CB22 3AT, UK; ^5^European Molecular Biology Laboratory, European Bioinformatics Institute, Wellcome Genome Campus, Hinxton, Cambridge CB10 1SD, UK; ^6^Pfizer Inc, 10646 Science Center Dr, San Diego, CA 92121, USA; ^7^Institutionen för cell-och molekylärbiologi, Uppsala University, Husargatan 3, Uppsala 752 37, Sweden; ^8^CeMM Research Center for Molecular Medicine of the Austrian Academy of Sciences, Vienna a-1090, Austria; ^9^Genentech Inc, 1 DNA Way, South San Francisco, CA 94080, USA; ^10^The Human Genome Sequencing Center, Baylor College of Medicine, Houston, TX 77030, USA

## Abstract

The Ensembl gene annotation system has been used to annotate over 70 different vertebrate species across a wide range of genome projects. Furthermore, it generates the automatic alignment-based annotation for the human and mouse GENCODE gene sets. The system is based on the alignment of biological sequences, including cDNAs, proteins and RNA-seq reads, to the target genome in order to construct candidate transcript models. Careful assessment and filtering of these candidate transcripts ultimately leads to the final gene set, which is made available on the Ensembl website. Here, we describe the annotation process in detail.

Database URL: http://www.ensembl.org/index.html

## Background

Sequenced genomes represent an extremely useful resource in biological research. High quality annotations maximize the utility of these genomes, as it is the annotations that link genomic sequence to biological function. The ultimate aim of genome annotation, therefore, is to identify the functional elements within a genome sequence such as the regions that are transcribed into mRNA, as well as those involved in regulation and expression.

Ensembl provides high quality integrated genomics resources for publicly available vertebrate genome assemblies. Since the project was launched 16 years ago (1), our gene sets have maintained a reputation as being of the highest quality (2, 3). Apart from being major components of the GENCODE (4, 5) gene sets, our annotations have also been the primary annotations used in the initial genomic analyses for a number of genome projects (Table 1). Furthermore, they have been used in a plethora of research disciplines across the array of species for which we provide annotations. Such examples include, but are not limited to, studies of disease (6–9), vertebrate evolution and divergence (10–14), metabolism (15) and gene expression (16). The extensive reuse of Ensembl gene sets in these and other studies, combined with experience and continual development in genome annotation, has established Ensembl as an authority in vertebrate genome annotation (17, 18).

**Table 1.** Genome projects for which Ensembl provided the primary annotation

Common name	Scientific name	Assembly name in Ensembl	Assembly accession	Ensembl release number	Ensembl release date	References
Duck	*Anas platyrhynchos*	BGI_duck_1.0	GCA_000355885.1	73	September 2013	(19)
Anole lizard	*Anolis carolinensis*	AnoCar2.0	GCA_000090745.2	61	February 2011	(20)
Cave fish	*Astyanax mexicanus*	AstMex102	GCA_000372685.1	74	December 2013	(21)
Cow	*Bos taurus*	UMD3.1	GCA_000003055.3	64	September 2011	(22)
Dog	*Canis lupus familiaris*	CanFam3.1	GCA_000002285.2	68	July 2012	(23)
Zebrafish	*Danio rerio*	Zv9	GCA_000002035.2	60	November 2010	(24)
Horse	*Equus caballus*	Equ Cab 2	GCA_000002305.1	49	March 2008	(25)
Atlantic cod	*Gadus morhua*	gadMor1	GCA_000231765.1	65	December 2011	(26)
Chicken	*Gallus gallus*	Galgal4	GCA_000002315.2	71	April 2013	(27)
Stickleback	*Gasterosteus aculeatus*	BROAD S1	GCA_000180675.1	40	August 2006	(28)
Gorilla	*Gorilla gorilla gorilla*	gorGor3.1	GCA_000151905.1	63	June 2011	(29)
Coelacanth	*Latimeria chalumnae*	LatCha1	GCA_000225785.1	66	February 2012	(30)
Rhesus macaque	*Macaca mulatta*	MMUL 1.0	n/a	40	August 2006	(31)
Wallaby	*Macropus eugenii*	Meug_1.0	GCA_000004035.1	55	July 2009	(32)
Turkey	*Meleagris gallopavo*	Turkey_2.01	GCA_000146605.1	61	February 2011	(33)
Opossum	*Monodelphis domestica*	monDom5	GCF_000002295.2	44	April 2007	(34)
Mouse	*Mus musculus*	GRCm38	GCA_000001635.6	68	July 2012	(35)
Ferret	*Mustela putorius furo*	MusPutFur1.0	GCA_000215625.1	69	October 2012	(36)
Gibbon	*Nomascus leucogenys*	Nleu1.0	GCA_000146795.1	63	June 2011	(37)
Nile tilapia	*Oreochromis niloticus*	Orenil1.0	GCA_000188235.1	67	May 2012	(38)
Platypus	*Ornithorhynchus anatinus*	OANA5	GCF_000002275.2	42	December 2006	(39)
Sheep	*Ovis aries*	Oar_v3.1	GCA_000298735.1	74	December 2013	(40)
Lamprey	*Petromyzon marinus*	Pmarinus_7.0	GCA_000148955.1	64	September 2011	(41)
Orang-utan	*Pongo abelii*	PPYG2	GCA_000001545.1	49	March 2008	(42)
Rat	*Rattus norvegicus*	Rnor_5.0	GCA_000001895.3	70	January 2013	(43)
Tasmanian devil	*Sarcophilus harrisii*	Devil_ref v7.0	GCA_000189315.1	64	September 2011	(44)
Pig	*Sus scrofa*	Sscrofa10.2	GCA_000003025.4	67	May 2012	(45)
Zebra finch	*Taeniopygia guttata*	taeGut3.2.4	GCA_000151805.2	53	March 2009	(46)
Platyfish	*Xiphophorus maculatus*	Xipmac4.4.2	GCA_000241075.1	71	April 2013	(47)

Species are listed along with assembly names, assembly accessions, the Ensembl release numbers where the annotations were first made available, the dates of these Ensembl releases, and the references for each of the respective published genome projects. The rhesus macaque assembly (MMUL 1.0) was published before the Browser Genome Release Agreement came into effect and therefore was not assigned an assembly accession.

The Ensembl gene annotation system is used for all vertebrate species in Ensembl. When providing gene annotation on a genome assembly, our main goal is to identify a set of full-length protein-coding genes. High accuracy, as judged by community assessments, is achieved by a well-established core data flow that integrates alignments of expressed protein, cDNA and other biological sequences (48). All Ensembl transcript models are supported by experimental sequence evidence; none are predicted solely by *ab initio* methods.

Manual curation involves the evaluation of biological sequences aligned to the genome in order to support gene structures. The evidence for each gene structure is assessed by an individual who is trained in genome biology, and results in low throughput gene annotation that is especially valuable in biologically complex regions of the genome. Ensembl’s approach is to automate the decision-making steps followed by manual curators, as much as they can be, using the same alignments. High-throughput annotation is achieved because thousands of genes can be annotated in parallel. The main strengths of the Ensembl annotation methods are the speed and consistency with which genome-wide annotation can be provided to the research community. These advantages will become ever more important as the number of assembled genomes and the amount of data available for each species increase due to new sequencing technologies (49, 50).

The Ensembl gene annotation system described by Curwen *et al.* (48) was designed to annotate species with high-quality draft genome assemblies, where same-species protein sequences and full-length cDNA sequences were available as input for identifying many of the protein-coding genes. More recently, fragmented genome assemblies have become available for annotation, as have assemblies with limited availability of same-species protein or full-length cDNA sequences. For many species, RNA-seq is an additional data source available for gene annotation. To address these new challenges, our system has been extended to include methods for fast and effective annotation of assemblies that are fragmented and for which there are relatively small amounts of same-species data. Novel methods have been developed to use data from new sequencing technologies and to improve accuracy for high-coverage genomes. We will give a general overview of our gene annotation (genebuild) process, and discuss the pipelines used within each phase. We will also highlight changes with respect to the process described by Hubbard *et al.* (51) and Curwen *et al.* (48), and introduce new methods that have since been added. Brief descriptions of how these processes have been applied to annotate the mouse, Tasmanian devil and chimpanzee genomes can be found in the Supplementary Information.

## Results

**Figure 1.**
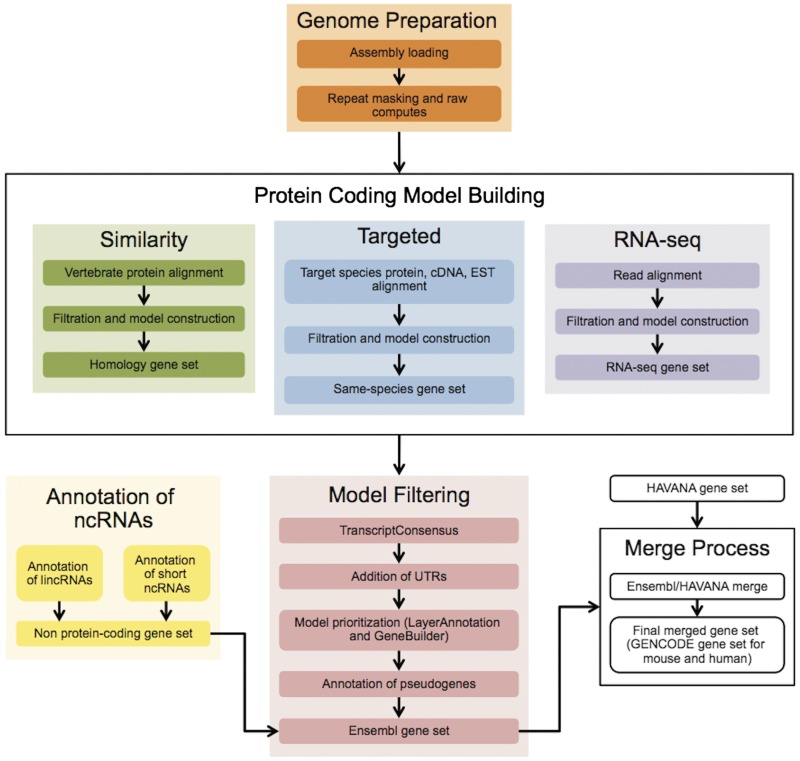
The Ensembl Genebuild workflow for annotating genes. The first phase of the annotation process is the Genome Preparation stage, which prepares the genome for gene annotation. The second phase is the Protein-coding Model Building stage, consisting of the Similarity, Targeted and RNA-seq pipelines. This generates a large set of potential protein-coding transcript models by aligning biological sequences to the genome and then inferring transcript models (exon–intron structures) using the alignments. Noncoding genes are annotated separately. Usually, the final phase is the Model Filtering stage. This involves sorting through the potential coding transcript models and filtering out those that are not well supported. Pseudogenes are then annotated and the noncoding RNA genes are incorporated to create the Ensembl gene set, which is then cross-referenced with external data sources. For some species (human, mouse, rat, zebrafish and pig) the HAVANA group produces manually curated gene sets. These annotations are merged with our Ensembl gene set to produce the final merged gene set. In the case of mouse and human, the merged sets comprise the GENCODE sets of genes.

The Ensembl gene annotation process (Figure 1) can be divided into four main phases: Genome Preparation, Protein-coding Model Building, Filtering and Gene Set Finalization. Each stage is described below, along with a selection of new methods. We also describe methods for post-release updates to a gene set.

Some of the methods described are required for every genebuild, whereas others are optional and can be employed to improve the gene annotation wherever necessary. The choice of process is influenced by the position of the species on the phylogenetic tree, the assembly quality and the availability of same-species protein and cDNA sequence data (see Supplementary Information). The Results section will detail recent changes and improvements to this system.

## Genome preparation

Ensembl does not produce genome assemblies. Instead, we provide annotation on genome assemblies that have been deposited into a member database of the International Nucleotide Sequence Database Consortium [INSDC: GenBank (52), ENA (53) and DDBJ (54)] and are therefore publicly available. We select species to annotate on a case-by-case basis according to a number of factors such as phylogenetic position, assembly quality, value of the organism as a disease model, availability of same-species sequence data (e.g. RNA-seq) and additional funding. For some species more than one genome assembly has been produced. In these cases Ensembl, NCBI and UCSC consult the species’ community in order to determine which assembly should be annotated as reference.

Once we obtain an assembly from one of the INSDC repositories, we load it into a database and prepare it for sequence alignment by running the repeat masking and raw compute analyses (Figure 1). The steps contained in this ‘Genome Preparation’ stage are followed for every genebuild.

### Assembly loading

**Figure 2.**
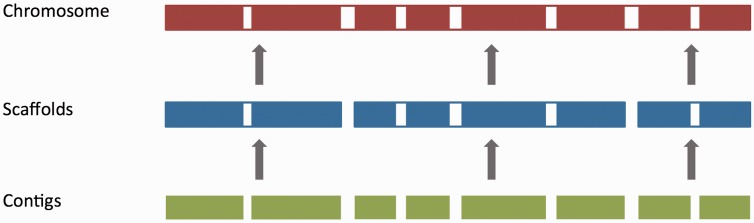
The genome assembly. Vertebrate genome assemblies usually comprise a number of possible layers of information. In most cases, sequenced reads will be assembled into contigs. Contigs are assembled into scaffolds based on linkage data (e.g. paired reads, or markers), and these scaffolds may be assembled to produce chromosomes.

For vertebrate genome assemblies, assembly loading usually involves inserting a list of contig (component), scaffold and chromosome accessions (where available) into an Ensembl core database schema (55). Contigs are the basic unit of a hierarchical genome assembly, with each contig comprising contiguous sequence with no gaps. Mate-pairing information is used to link contigs together into longer structures called scaffolds, and scaffolds may be linked together to form chromosomes (Figure 2). Each of these levels has its own coordinate system that is relative to the start of the sequences on that level.

DNA sequences for all the contigs are first stored in the database. We then load mappings between each coordinate system, using the AGP (‘A Golden Path’) files provided with the assembly. These files identify equivalent regions between sequences on different coordinate systems. We load contig-to-scaffold, contig-to-chromosome and scaffold-to-chromosome mappings. It is not necessary to store DNA for scaffold or chromosome sequences as these higher level structures can be constructed from their component contig sequences. Next, we label as ‘toplevel’ the sequences in the genome assembly that are not a component of another sequence region. Toplevel is therefore a virtual coordinate system that includes the available chromosome sequences, as well as all the scaffold sequences that are not placed within a chromosome. All gene annotation processes are run across the toplevel coordinate system (56).

### Repeat masking

RepeatMasker (57), Dust (58) and Tandem Repeat Finder (59) (TRF) are used to mask repetitive genomic sequence. RepBase repeat libraries (60) are used for RepeatMasker, and in 2011, we started to run RepeatModeler (61) to generate our own libraries for some species that are distantly related to the well-characterized mammals, such as coelacanth. When preparing a RepeatModeler library, we use BLAST (62) to align the repeat library output produced by RepeatModeler to the UniProt (63) protein existence (PE) level 1 and PE level 2 proteins. The UniProt PE levels indicate that there is experimental evidence for the protein or transcript, respectively. Any repeat sequence with a significant hit to UniProt is removed from the RepeatModeler library so as to minimize the number of repetitive protein sequences that will be included in the library for repeat masking.

Several RepeatMasker analyses may be run; one for each of various chosen RepBase libraries and one for the custom RepeatModeler library generated in-house. We then assess the results in order to select those RepeatMasker runs that maximize the proportion of the genome that is repeat masked, while also minimizing the number of repeat libraries used. Results from the remaining RepeatMasker runs are not used in subsequent analyses.

### Raw computes pipeline

‘Raw computes’ (56) is a collective term for the selection of primary annotation analyses that are run across the genome assembly immediately after repeat masking. The *ab initio* algorithms include Genscan (64), for predicting gene models; Eponine (65), for finding transcription start sites; CpG (Gos Micklem, unpublished software), for finding CpG islands; tRNAscan-SE (66), for finding potential tRNA genes and FirstEF (67), for identifying the first exons of transcripts. With the exception of Genscan (used for reducing the search space in the BLAST step described below), the results of these analyses are not used in the genebuild; they are run purely for website display purposes.

We also BLAST all of UniProt, UniGene and vertebrate RNAs from the ENA against the Genscan peptide sequences. Running this step across the Genscan results, rather than across the whole genome, reduces the compute time required. The result of the UniProt BLAST step is used later in the genebuild if the Similarity pipeline (described below) is run as part of the protein-coding gene annotation system.

## Protein-coding model building

The model-building phase involves the alignment of protein, cDNA, EST and RNA-seq sequences to the genome assembly. The methods used in this phase depend on the input data available at the time of annotation. Input datasets are selected taking provenance into account, with same-species data preferred over data from other species, and with annotated sequences preferred over computed sequences. The final output of this section of the genebuild is a collection of databases that contain sequence alignments and a large set of potential protein-coding transcript models.

### Targeted pipeline

The Targeted (same-species) pipeline uses same-species protein sequences to first identify the rough genomic location of protein-coding genes, and then to produce coding models using GeneWise (68). This two-step method aims to speed up the process by reducing the search space made available to GeneWise to a subsection of the genome, which has similarity to the protein sequence being aligned.

Same-species protein sequences are downloaded from UniProt and RefSeq (69), with the aim of restricting these to a set of high-confidence input sequences. For UniProt, we download only Swiss-Prot and TrEMBL protein sequences labeled as PE level 1 and PE level 2. In the case of RefSeq, we download sequences with ‘NP’ and ‘AP’ accessions, which are the annotated protein sequences. RefSeq computed protein sequences including the ‘XP’ accessions are not downloaded. The combined set of downloaded UniProt and RefSeq protein sequences form the input for the Targeted pipeline.

We locate the approximate genomic location of transcripts by aligning protein sequences to the genome using Pmatch (R. Durbin, unpublished software) with a threshold of ‘-T 14’. This threshold indicates the number of consecutive amino acids that must exactly match the genomic DNA, and is an efficient method for aligning proteins when they have high identity to the genome. It is important not to lose too many same-species input sequences at this early stage of the genebuild process. Thus, if Pmatch does not align all input proteins, we then align the remaining protein sequences using Exonerate (70).

Every Pmatch hit will correspond to translated exonic sequence. Pmatch hits from each input protein sequence are grouped along the lengths of genomic sequences, using the module [also referred to as a Runnable (56)] BestPmatch, so that the genomic range of the hits roughly corresponds to the location of the input protein’s transcript. The genomic range identified by BestPmatch is extended by 200 kb in both directions and the DNA sequence for this region is passed to GeneWise, along with the original input protein sequence. GeneWise aligns the protein sequence to the DNA using a splice-aware algorithm and generates a protein-coding transcript model as output.

For human, mouse and selected other species, we run GeneWise at least twice across the genome: a first time requiring consensus splicing and a second to allow nonconsensus splice sites. While consensus splicing is more common than nonconsensus splicing, the second run of GeneWise provides flexibility for those coding models with real nonconsensus splice sites and permits alignment of the protein sequence in regions where there are genomic sequence errors. Some models produced by GeneWise contain small ‘frameshift introns’ of 1, 2, 4 or 5 bp long where errors, insertions or deletions in the genomic sequence would otherwise introduce translation frameshifts. When translated off the genomic sequence, the coding sequence for these models is more likely to be full length, which is particularly useful in lower quality draft genomes.

In Curwen *et al.* (48), we described passing ‘MiniSeqs’ to GeneWise. However, we no longer use this approach. We now use ‘FullSeqs’ that include all genomic sequence from the first to last Pmatch alignments; intronic genomic sequence is no longer removed. This FullSeq method is possible due to increased computational resources and optimization of the GeneWise program. It is preferred because it allows GeneWise to search the full genomic sequence and to correctly place short exons, while genomic sequences for short exons were not always present in the MiniSeqs.

In addition to GeneWise, we also use Exonerate’s cdna2genome tool (70) to generate protein-coding gene models. This is achieved by downloading cDNA sequences that have a coding sequence (CDS) range annotated in the INSDC record; cDNA sequences without an annotated CDS in the INSDC record are not used in this step. Combined alignment of a cDNA and its annotated CDS by Exonerate has the advantage of adding untranslated regions (UTRs) to the protein-coding models in one step, and of ensuring that the correct UTR is added to a coding model. This step is only run for the handful of species that have large numbers of annotated protein-cDNA pairings. As Exonerate produces models whose translation include stop codons, we search each of the resulting models and remove those with more than one internal stop. For models with only a single internal stop codon, a small frameshift intron is introduced in its place.

From the multiple GeneWise and Exonerate methods described above, each original protein sequence may have produced multiple coding transcript models at one location, with slightly different exon structures and translated sequences, depending on the degree to which the protein sequence matches the genome. In order to identify the model whose translation most closely matches the input sequence, the translation from each of these models is aligned back to the original protein sequence by the BestTargeted module, using Exonerate’s ‘affine:local’ model. This is a local alignment that uses the affine gap penalty, similar to the Smith–Waterman–Gotoh algorithm (71). For each original protein sequence, the Ensembl model producing the highest Exonerate score is selected to be the final output for the Targeted pipeline.

### Similarity pipeline

As with the Targeted pipeline, the aim of the Similarity pipeline is to identify the rough genomic location of protein-coding transcripts and then to produce coding models using GeneWise. Unlike the Targeted pipeline, which restricts its input to only same-species proteins, the Similarity pipeline takes as input UniProt proteins from a wide range of species. This approach is especially useful for species that do not have many same-species proteins suitable for use in the Targeted pipeline such as elephant or anole lizard, but is less so for well-described species with many proteins in UniProt, such as human and mouse.

The method for reducing the genomic search space passed to GeneWise is different in the Similarity pipeline compared to the Targeted pipeline. Instead of using Pmatch to identify the rough placement of protein sequences, we use the UniProt BLAST results produced in the raw compute pipeline. Although BLAST requires more compute resource than Pmatch to run, it is more tolerant of the sequence mismatches that typically occur when aligning proteins from the broad range of species used in the Similarity pipeline.

The UniProt BLAST results are first classified across three axes according to the information provided by UniProt: by PE level, by source (Swiss-Prot or TrEMBL) and by taxonomy. This division of UniProt subsets allows us to prioritize the reviewed protein sequences that are more closely related to the species being annotated.

UniProt proteins that mapped to a Genscan peptide sequence during the raw computes step are then aligned to the full genomic sequence underlying the Genscan model, again using BLAST. This step allows hits to be identified outside of the Genscan exons. It is these results that define the regions on which GeneWise is subsequently run.

The output of the Similarity pipeline is a set of models, based on protein sequences from a variety of species, which supplements the models already generated by the Targeted pipeline.

### RNA-seq pipeline

With the rapid adoption of high-throughput transcriptome sequencing (i.e. RNA-seq) as an experimental method, the amount of available transcribed sequence data is increasing dramatically (72). The quality of such sequence data is expected to continue to increase over the next few years, making it a valuable resource in the gene annotation process.

The main difficulty in using short reads for gene annotation is that the full length of an mRNA is not represented in one contiguous sequence. These short sequences must be combined to generate longer transcript models without full knowledge of the splicing pattern of the exons in each expressed isoform. The paired reads provide more informative alignments than single reads because reads that align as a pair have a higher confidence level of being aligned correctly (73). It is also possible to take the expected insert size for paired reads into account when validating their alignments. Stranded reads are particularly useful for cases in which transcripts overlap on opposite strands, and assignment of a read to the correct strand can be ambiguous, although for un-stranded reads, a transcript’s strand can normally be determined from the direction of splice sites. Most of the RNA-seq data with which we have worked have been paired-end reads of 50 bases or longer, generated by Illumina machines.

Because short read data do not allow the confident construction of full-length splicing models, the Ensembl RNA-seq pipeline is usually configured to produce only one transcript model per gene as output. This conservative approach aims to prevent the introduction of false transcript structures that result from incorrectly combining exons and introns along the length of a model.

RNA-seq-based models are produced from a two-step alignment process with only minor modifications to that described by Collins *et al.* (74). Firstly, raw reads are now aligned to the genome using BWA (75). These alignments are collapsed to create alignment blocks that roughly correspond to transcribed exons. Read pairing information is then used to group putative exons into approximate transcript structures called proto-transcripts. In the second alignment step, the reads that were partially aligned by BWA are extracted and aligned to the proto-transcripts, or more commonly to the underlying genomic sequence, using Exonerate. Exonerate is splice-aware, providing alignments that allow us to infer introns. Finding clear exon–intron junctions is a challenge when the raw reads have been sequenced from a mixture of fully processed and partially processed transcripts; reads sequenced from retained intronic sequence can lead to the annotation of one long, false exon that should have been annotated as one intron surrounded by two exons. These false exons are removed when detected; they are identified by searching within the genomic range of each putative exon for evidence of spliced reads. The result of the Exonerate alignment step is a set of spliced alignments representing canonical and noncanonical introns. Transcript models are created by combining the transcribed regions from the proto-transcripts with the observed (intronic) spliced alignments to create all possible transcript isoforms indicated by the aligned data. We usually configure the system to only keep the isoform with the most read support across its splice junctions and exons.

Read length and depth of coverage are both important when identifying introns. When read coverage is high, it is more likely that the set of raw reads contains sequences that can be aligned across an intron. When reads are longer, it is more likely they will span an intron. Having reads that align across every intron in a transcript makes it possible for us to build a complete transcript model. If the coverage is very low, some splice boundaries may not be covered by a read in the raw data set. Without read support, these introns will not be generated in the Exonerate step, which can result in fragmented models or models with retained introns.

The RNA-seq pipeline produces both protein-coding and noncoding transcript models. The final step in this process is to BLAST UniProt PE 1 and PE 2 proteins against the set of RNA-seq models so as to identify the protein-coding transcript models. Our standard thresholds for the UniProt alignments are 80% identity and 80% coverage of the sequences.

For the reads from each input sample, and for the merged set of reads from all samples, the output of the RNA-seq pipeline includes an indexed BAM file of the reads aligned by BWA, a set of intron features produced by aligning intron-spanning reads with Exonerate, and a set of transcript models. These data can be viewed as separate tissue tracks in the Ensembl browser. They can also be obtained through a programmatic interface.

Transcript models are produced separately for each of the tissue samples, as well as for the merged set. Transcript models from a single tissue input sample are often more fragmented than transcript models from the merged set. (The data in the merged set are deeper, and this allows more splice junctions to be detected and therefore more consecutive exons to be joined to produce longer models.) For this reason, transcript models resulting from typically only the merged set of reads are used for incorporating into the final gene set.

Intron features from the set of merged reads are used later on in the annotation process by the TranscriptConsensus module to filter Similarity models (described below). Transcript models from the set of merged reads may be used for adding UTRs to Targeted and Similarity models, and may also be included as part of the main gene set during the LayerAnnotation pipeline (also described below).

### Ortholog recovery pipeline

In preparing a set of preliminary transcript models produced by the model-building pipelines, comparative data may be used for both assessing the completeness of the transcript set and for supplementing the transcript set where appropriate. Transcript structures may be absent from a preliminary set for a number of reasons, most commonly because the genomic sequence is missing from the assembly or because the Targeted and Similarity pipelines did not produce a model. For the latter case, it may still be possible to annotate models using our ortholog recovery pipeline. The RNA-seq pipeline described above will also identify genes not found by the Targeted and Similarity pipelines, and so use of the ortholog recovery pipeline has become less common since RNA-seq data became more widely available.

The OrthologueEvaluator module was developed to identify and annotate additional transcript models based on orthology. OrthologueEvaluator takes as input the preliminary transcript set with the gene sets from at least two well-annotated species, usually human and mouse. A set of orthology predictions is generated by best reciprocal BLAST hits across the input sets. These predictions are then used to fill in gaps and to supplement truncated models. In both cases, the Ensembl protein sequence of an ortholog from one of the well-annotated species is selected for alignment, with Exonerate, to the genome being annotated. When Exonerate generates a good alignment the resulting model is added to the preliminary transcript set.

### Projection pipeline

The Targeted and Similarity steps rely on the alignment of complete protein sequences to the genome sequence. This method is unsuitable for low-coverage fragmented assemblies where missing genomic sequence, mis-orientations and misplacements occur more frequently than in the higher quality draft genome assemblies. In fragmented assemblies many genes will be represented only partially (or not at all) in the assembly, and many others (particularly those genes with large genomic extent) will be found in pieces, distributed across more than one scaffold.

**Figure 3.**
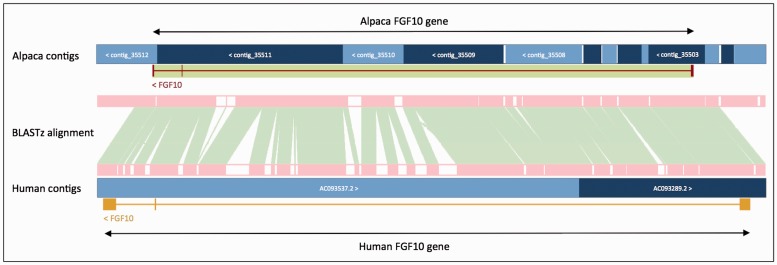
Projection of human *FGF10* to alpaca. The *FGF10* gene in alpaca was annotated by aligning the human and alpaca assemblies using BLASTz, and then projecting (copying) the human gene onto the alpaca genome. A novel structure, GeneScaffold_2975, was generated in the alpaca assembly by bringing together the shorter scaffolds that aligned to the human region containing the *FGF10* gene.

In order to improve gene annotation on species with fragmented assemblies, we developed a methodology that relies on a whole genome alignment (WGA) to an annotated reference genome—usually the human genome. This method was used, as follows, to annotate all of the low-coverage mammal genomes produced by the 29 Mammals Project (76). For each of the low coverage target genomes, the whole-genome alignment between the human genome and target was generated using BLASTz (77). The resulting set of local alignments was linked into chains using axtTools (78). A custom filter was then applied to ensure that each base pair in the target genome aligned to no more than one position in the human genome. The WGA block underlying each annotated gene structure in the human genome was used as a guide to bring together scaffolds from the target species and join them into longer ‘GeneScaffolds’ (Figure 3) that could contain complete gene structures. The inferred GeneScaffolds created a virtual assembly on top of the target species’ primary assembly. Genes from the human genome were then ‘projected’ (copied) down on to the target genome. In regions where the WGA implied that the target assembly was missing genomic sequence containing an internal exon, the projected exon was placed on the gap sequence. This resulted in a string of Xs corresponding in length to the projected translation. The creation of GeneScaffolds altered the set of toplevel sequences that were initially loaded into the Ensembl database, so the raw compute analyses were run across the new GeneScaffolds. This method of altering the toplevel sequences is no longer used because it would hinder navigation between Ensembl and other genome browsers such as UCSC and NCBI.

This method of whole-genome alignment and projection of annotation from the human genome to the target assembly was also applied to higher primates. However, the creation of GeneScaffolds was unnecessary because the primate assemblies were of better quality or were created using order and orientation information from the human assembly.

### Extending protein-coding models into their UTRs

Protein-coding models generated from protein-to-genome alignments in the Targeted, Similarity and Ortholog recovery pipelines will not have UTRs annotated. Targeted models produced by Exonerate’s cdna2genome model, on the other hand, do not require UTR extension because they are based on the alignment of cDNA and will already have UTRs annotated.

Models made from RNA-seq, cDNA or EST sequences can be used to add UTRs to the coding models. We have already described the RNA-seq pipeline and how these models are generated. For cDNAs, models are generated by aligning the cDNA sequences to the softmasked genome using Exonerate. ESTs are aligned in the same way as cDNAs, and these alignments are collapsed into models using the EST2genes or TranscriptCoalescer modules. These two modules combine spliced EST alignments into longer transcript structures.

The variable quality of EST data, which often come from multiple labs using different protocols, makes the sequences difficult to incorporate into an annotation system that expects data to be of a consistently high quality. We do not use EST models for UTR addition unless a species has a large number of EST sequences and very little cDNA or RNA-seq data.

The UTR_Builder module traverses each toplevel sequence and identifies protein-coding models that are overlapped by RNA-seq, cDNA or EST models. When the start and end boundaries of the first intron of a protein-coding model are matched by an RNA-seq, cDNA or EST structure, this sequence evidence can be used to add a UTR at the 5-prime end. The same rule applies to the last intron of a protein-coding model when adding the 3-prime UTR. For single-exon transcripts, the exon start and end must lie within the corresponding sequence evidence in order to add a UTR. When a translation does not start with a Methionine, the UTR is searched upstream of the CDS for the first in-frame Methionine. Similarly, when a translation does not end in a stop codon the UTR is searched up to 150 bases downstream of the CDS for the first in-frame stop codon.

CAGE (79) and paired-end tags (ditags) (80) provide information on the transcription start and end positions. We have adapted our UTR pipeline to make use of these data so as to define UTR boundaries more precisely. The genomic locations of CAGE tags and ditags are compared against the cDNA models, which allows scoring of each potential pairing of protein model to cDNA. The UTR_Builder module prioritizes the cDNA model with the most CAGE and ditag support. This has been applied in human and mouse where deep sequencing data are available.

**Figure 4.**
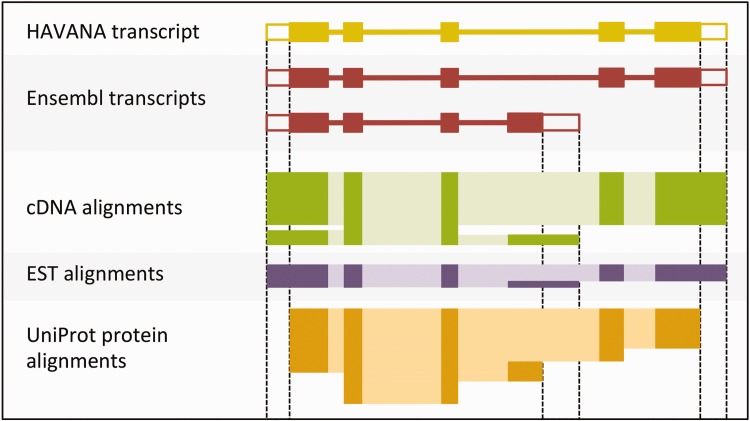
Sample transcript models with supporting evidence for untranslated regions (UTRs). This figure shows sample transcript models from HAVANA (yellow) and Ensembl (red) aligned with supporting evidence from cDNAs (green), ESTs (purple) and proteins (orange). Darker colors in the alignments correspond with exons. Unfilled boxes at the ends of the transcripts represent UTRs. Support for the UTRs comes from the aligned cDNAs and ESTs but not from the proteins.

The output of the UTR_Builder step is an updated set of protein-coding transcript models that have been extended to include UTRs where evidence was available (Figure 4). The cDNA and EST models are used in filtering steps later on and are also displayed on the website along with the ESTgenes.

### Special types of protein-coding genes

The protein-coding gene annotation process described above creates high quality gene models throughout most of the genome. The annotation process relies on aligning protein sequences to the genome and is suitable for most protein-coding genes.

There are certain types of protein-coding genes, however, where the above approach is not suitable. These include Immunoglobulin/T-cell receptor genes and selenoproteins. We have developed separate approaches to improve annotation for both such cases.

#### Immunoglobulins and T-cell receptors

The Immunoglobulin/T-cell receptor clusters are difficult to annotate because the underlying genomic region undergoes somatic recombination. This process of genome rearrangement combines multiple genes from the cluster—known as Variable (V), Constant (C), Diverse (D) and Joining (J) genes—by excising the intervening DNA. This generates a functional immunoglobulin gene sequence that encodes a complete immunoglobulin/T-cell receptor.

We aim to annotate the individual V, D, J and C genes. However, many records of proteins in UniProt and cDNAs in ENA are full-length products of transcripts expressed after the associated V(D)J somatic recombination events. Each of these records contains sequence for multiple genes, which would need to be separated to generate the correct annotation.

The V, D, J and C gene boundaries are often incorrectly predicted when aligned back to the un-rearranged reference genome using a spliced-alignment program such as GeneWise or Exonerate. This is because the junctions are not generated by the standard splicing machinery, and therefore do not display the standard splicing signals.

Annotation for T-cell receptors and immunoglobulin genes has been improved for human and mouse by collaborating with other annotators who contribute to the International Immunogenetics information system (IMGT) (81). This database contains annotations of individual genes on RNA and genomic DNA reference entries. The IMGT genes are aligned to the genome using Exonerate and are then merged with our gene annotations. Existing transcript models that overlap at the exon level with the aligned IMGT genes are removed.

#### Selenoproteins

Selenocysteines are encoded by UGA, one of the three codons responsible for translation termination. To represent these codons as encoding selenocysteines instead of stop codons, we align UniProt records with the ‘SEL_CYS’ tag to the genome using Exonerate. The stop codons at the relevant positions specified by these records are then replaced with selenocysteine residues.

## Model filtering

The aim of the model filtering phase is to determine a subset of protein-coding transcript models, generated by the different model-building pipelines, that will comprise the final protein-coding gene set.

Although we are careful to select input protein sequences that are of high confidence and from species closely related to the target genome, the model-building phase creates models that result from poor protein alignment and must be removed. The annotated splicing structures for these models may be unique but they are not biologically real alternate isoforms. While the model-building phase has an emphasis on sensitivity, where we align large numbers of sequences to the genome with a broad range of alignment thresholds, the model filtering phase has an emphasis on specificity and will select only the models with the highest confidence at each locus to take forward to the final gene set.

### TranscriptConsensus

TranscriptConsensus is a filtering module that is run routinely for all genebuilds with the aim of removing putative alternate transcript isoforms that are not well supported. The poorly supported models are most likely to arise from a poor protein alignment in the Similarity pipeline: when proteins from distantly related species are aligned to the genome and used as evidence to annotate a model, the low identity match between the protein and the genome can confound the GeneWise alignment and result in a model with a poorly supported splicing structure. TranscriptConsensus compares the protein-coding models produced by the Similarity pipeline against available same-species evidence: cDNA, EST and RNA-seq models, and RNA-seq introns.

In this module, each protein-coding model from the Similarity pipeline is scored by comparing its exon and intron boundaries to those of the same-species cDNA, EST and RNA-seq models. The scoring of each candidate model is weighted by the length and score of other overlapping protein-coding models. The highest scoring models will be labelled as ‘good’ and low scoring overlapping models will be labelled ‘bad’ and excluded. We typically require a depth of at least six same-species models in order to score and distinguish between the good and bad models. When too few cDNAs are available to determine the score of the protein-coding model it is labeled as ‘small’. The output of this step is a classification of the set of protein-coding transcript models according to how well their exons and introns are supported.

### LayerAnnotation

**Figure 5.**
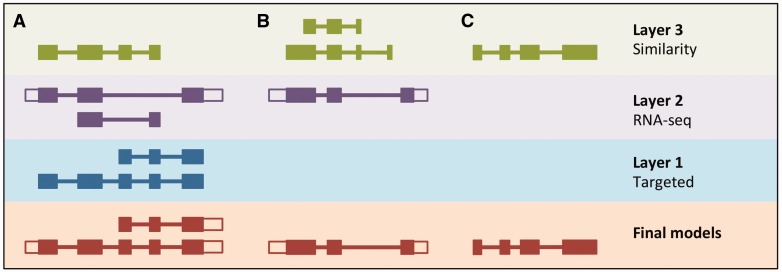
LayerAnnotation method. Candidate transcript models produced by each of the model-building pipelines are assigned varying levels of priority. In this example, models produced by the Targeted pipeline (which uses same-species protein data) are placed in Layer 1 and are therefore given preference over models with overlapping exons from the other model-building pipelines. Models produced using RNA-seq data are placed in Layer 2 and are given priority over those produced by the Similarity pipeline (which uses protein data from other species) in Layer 3. Final models indicate those selected for the final Ensembl gene set. (**A**) Candidate transcript models were produced by three model-building pipelines. The final protein-coding models were selected from Layer 1. Untranslated regions (unfilled boxes) were added from an RNA-seq model in Layer 2. The two transcript models will later be collapsed into a single gene model. (**B**) Layer 1 contains no model that overlaps with the model in Layer 2, and so the model in Layer 2 is the final model. (**C**) Layer 1 and Layer 2 contain no models that overlap with that in Layer 3, so the model in Layer 3 is selected as the final one.

The LayerAnnotation module allows us to define a hierarchy of input sets of models, from most preferred to least preferred, and to selectively filter out models in the less preferred input sets (Figure 5).

All candidate protein-coding models are used as input for LayerAnnotation. Models supported by same-species data (Targeted and RNA-seq) are prioritized in the top layer of the hierarchy and will all be selected. Where there are few models supported by same-species data, these models will not contribute significantly to the final gene set. Next, we rank transcript models generated from the protein sequences of species with a relatively short evolutionary distance to the species being annotated. This information comes from the classification of the Similarity transcript models according to their taxonomic position. We assume also that we should prioritize transcript models that have their exon–intron structure well supported by other sources of sequence alignment such as cDNAs and ESTs. This information comes from the classification of models according to PE level and the TranscriptConsensus module (‘good’, ‘small’ or ‘bad’). The ‘good’ Similarity models are inserted into the hierarchy in successive layers that represent increasing evolutionary distance, followed by the ‘small’ Similarity models. Models from lower layers in the hierarchy will only be selected when they do not overlap models from the preferred layers.

For example, when annotating the rat genome, we might choose the following hierarchy: first, the models supported by rat data from the Targeted and RNA-seq pipelines; second, the murine models from the Similarity pipeline that are labeled as ‘good’ by TranscriptConsensus; third, the remaining models labeled as ‘good’ by TranscriptConsensus.

For every species, the relative contribution from each of the model-building pipelines to the final gene set will vary depending on the priority given to each set of models and the number of models in each of the sets. When models produced by same-species data are available, we heavily prioritize their inclusion over the homology-based ones. For well-studied species, the Targeted pipeline will contribute the majority of gene models to the final gene set. The Similarity pipeline contributed the bulk of gene models for most other vertebrate species prior to RNA-seq data becoming widely available. For more recent genebuilds, the primary source of data is now same-species RNA-seq supported by other-species protein alignments, with Similarity from other species as the next major source of gene models. Detailed information on these relative contributions can be found on the Ensembl species home pages by clicking on the link that says ‘More information and statistics’.

### GeneBuilder

The transcript models selected by LayerAnnotation are passed to the GeneBuilder module. The aim of this module is to remove redundant transcript models and produce multi-transcript, protein-coding genes.

GeneBuilder clusters protein-coding models into multi-transcript gene structures when their coding exons overlap. It will then remove those models where the splicing pattern is completely redundant (i.e. no unique splices) when compared to a longer model.

### Pseudogenes

All of the gene models produced by the GeneBuilder module are labeled as protein-coding because they are supported by aligned protein sequences. The Pseudogene annotation method aims to identify processed pseudogenes from within this set of gene models and to label them accordingly.

Our annotation system allows protein sequences to align imperfectly to the genome. In most cases, this is advantageous because it allows models to be generated where there is genomic variation or a sequencing error. In some cases, a protein sequence may align to a pseudogenic region of the genome, resulting in a gene model that our annotation initially labels incorrectly as protein-coding.

Some protein sequences align to multiple regions in the genome, giving rise to multiple gene models. Where these multiple gene models are either all multi-exon or all single-exon, we assume that they belong to a functional gene family. However, there are cases where one protein sequence gives rise to both multi-exon and single-exon genes. Such cases suggest that a process of retrotransposition occurred, generating unspliced copies of the multi-exon gene in the genome, and the protein sequence has aligned to both the parent and pseudogenic copies. The single-exon gene models in these cases are consequently labeled by the Pseudogene module as ‘processed pseudogenes’.

In addition, the Pseudogene module searches for protein-coding gene models that have a high proportion of their intronic sequence composed of repeats. This suggests that repetitive sequence was inserted into an otherwise single-exon region, which may have resulted in loss of function and pseudogenization. It also identifies models where all introns are fewer than nine bases long. These frameshift introns may indicate a degenerate coding region. In these cases, the gene models are labeled by the Pseudogene module as ‘pseudogenes’.

The output of the Pseudogene pipeline is a genome-wide set of gene models, with genes labeled as either protein-coding or pseudogene. For most species this is the final gene set. However, there are additional methods that may be run for selected species.

## Gene set finalization

This section describes optional methods that may be run after the Pseudogene module. These methods serve three main purposes: to add noncoding gene models to the gene set, to incorporate annotations from external groups, and to add additional information to the annotated genes. Each of these methods is discussed below.

### Annotation of nonprotein-coding genes

#### Short noncoding RNA pipeline

As with proteins, the structure of the noncoding RNA (ncRNA) molecule imparts biological function. However, while related proteins have conserved primary sequences this is not necessarily the case for ncRNAs. Therefore, our standard sequence alignment methods used for the annotation of protein-coding genes are not suitable for annotating small ncRNA genes (82).

In order to annotate small ncRNA genes, sequences from Rfam (83) are first aligned to the genome using BLASTN. Although the resulting alignments will over-predict the number of potential ncRNA loci, BLAST is a useful tool for narrowing genomic search space. Next, the Infernal suite of programs (84) filters the BLAST hits using a covariance model that incorporates information about the ncRNA secondary structure. Finally, Infernal’s cmsearch is used to build ncRNA models.

MicroRNAs (miRNAs) are annotated by an initial BLASTN search of genomic sequence against miRBase (85) stem-loop sequences, followed by filtering of the results according to E-value. Gene models are then created from these results when a folding calculation, performed by RNAFold (86), infers that the underlying genomic sequence forms a stable hairpin structure.

Transfer RNAs (tRNAs) are not annotated in this method. They are predicted during the raw compute pipeline, using the *ab initio* algorithm, tRNAscan-SE (66).

#### Long intergenic noncoding RNA pipeline

Long intergenic noncoding RNA (lincRNA) genes have a number of characteristics that make them a challenge to annotate (87). Our current method traverses each toplevel sequence and identifies models generated by the Exonerate alignments of cDNAs that do not overlap with protein-coding genes. Next, these candidate lincRNA models are compared against regions of chromatin methylation (H3K4me3 and H3K36me3) identified by the Ensembl Regulation pipeline (88). A final evaluation step determines whether or not each candidate lincRNA has protein-coding potential. Any candidate lincRNA containing a substantial open reading frame (ORF) (covering 35% or more of its length) and either Pfam or TIGRFAM (89) protein domains will be rejected. Candidate lincRNAs that pass the final evaluation step are included in the final Ensembl gene set as lincRNA genes. LincRNAs have, to date, only been annotated in this way for human and mouse.

### Incorporation of additional gene models from external sources

#### The Ensembl-HAVANA merge pipeline

**Figure 6.**
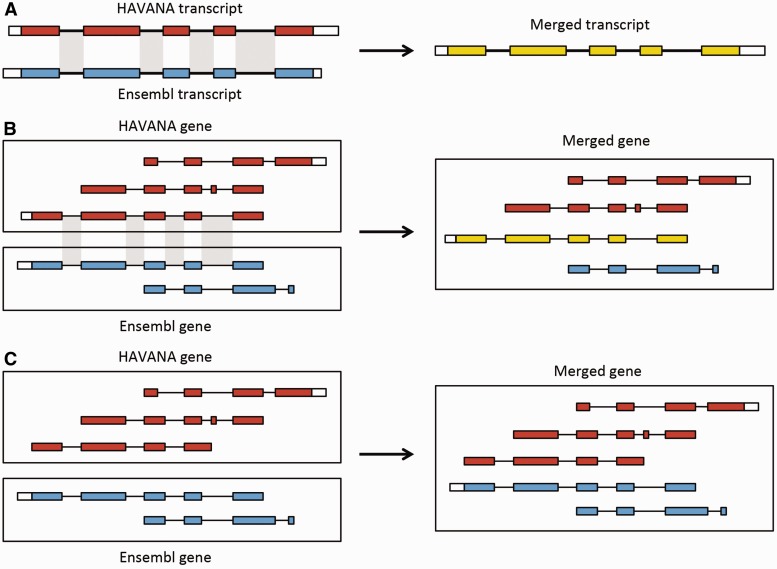
Merging gene and transcript models. For both Ensembl and HAVANA models, transcripts with overlapping exons are grouped together into genes. (**A**) If the intron–exon boundaries, excluding UTRs, of a transcript from HAVANA completely match those of one from Ensembl the result is a merged transcript model, which is always based on the HAVANA annotation. If the intron–exon boundaries do not completely match then the two models are treated as separate transcripts belonging to the same gene. (**B**) Exons for a HAVANA gene overlap with those for an Ensembl gene. All transcripts are grouped together in the same merged gene. The intron–exon boundaries for one HAVANA and one Ensembl transcript match perfectly so they are merged to create the merged transcript shown in yellow. (**C**) Exons for Ensembl and HAVANA transcripts overlap but there are no transcripts with complete matching intron–exon boundaries. We still group the transcripts together into a merged gene but no transcripts are merged.

The Ensembl-HAVANA merge pipeline combines the Ensembl annotation with the manually curated HAVANA set to produce a ‘merged’ gene set. The aim of this process is to create the most comprehensive gene set possible, by including the entire annotation from HAVANA and supplementing it with the Ensembl annotation (Figure 6). The Ensembl models fill the gaps where there are no HAVANA models, and they provide additional transcript isoforms using new sequence data that have not already been annotated. The full process has been described by Harrow *et al.* (4). This technique is only applied for human, mouse, zebrafish, rat and pig annotations. For human and mouse, the merged set of Ensembl and HAVANA genes form the GENCODE gene set (4).

#### Annotations from external sources

For some species other than human and mouse, external groups have produced their own gene annotations, which we have assessed and incorporated into the Ensembl gene set where appropriate. Currently, the platypus, zebra finch and anole lizard gene sets include selected models created in this way.

### CCDS

Ensembl collaborates in the Consensus Coding Sequence (CCDS) project (90, 91). This project provides a set of consistently annotated protein-coding gene models between GENCODE and RefSeq for human and mouse. When we update the gene models for either of these species, we ensure that all CCDS models are present by comparing our gene set against the latest snapshot of the CCDS tracking database. Any missing CCDS models are added back into the gene set before being released to the public.

### Additional annotations

At this point, the protein-coding and noncoding gene sets are finalized. The subsequent steps do not modify the gene models themselves, but rather add further information including stable identifiers, cross-references to external databases and positions of protein domains.

All genes, transcripts, translations and exons are assigned stable identifiers. When annotating a species for the first time, these identifiers are auto-generated. In all subsequent annotations for a species, the identifiers are propagated based on comparison of the new gene set to the previous gene set so that equivalent genes for a species can be discerned between releases. Stable identifiers have versions that are incremented when the sequence or coordinates of an exon, transcript or gene has been updated.

Genes, transcripts and translations are cross-referenced to external databases. This process adds gene names and descriptions where possible, and links the Ensembl annotations to entries in relevant resources. For human, this includes cross-referencing to the HGNC (92), RefSeq (69), UCSC (93), OMIM (94), CCDS, UniProt, ENA and other external databases. Transcripts with potential frameshift occurrences (exons 1, 2, 4 or 5 bp apart) are labeled in the database as having frameshift attributes.

Finally, Ensembl translations are scanned for protein signatures. We identify these signatures by both *ab initio* sequence search [SEG (95), SignalP (96), ncoils (97), TMHMM (98)] and searching protein domain databases for sequence matches [PRINTS (99), Pfscan (100), Pfam (101), TIGRFAM, SUPERFAMILY (102), SMART (103) and PIRSF (104)].

## Post-release updates to the gene set

Once the final gene set has been produced, it becomes part of a numbered Ensembl public release. For most species, the gene set remains stable for several releases and would not normally be updated until an improved assembly or significant new input data become available.

Minor updates to the protein-coding gene set are most likely to occur following genome-wide searches for gene models that are poorly supported. For example, models that do not have orthologs are investigated for removal.

There are currently four types of new data that may trigger a larger update to the gene set, which are outlined below.

### Noncoding RNAs

Noncoding RNAs are periodically updated to incorporate changes to the underlying Rfam and miRBase databases.

### HAVANA annotation

HAVANA is continuously adding to and updating their annotations on human, mouse, rat, pig and zebrafish. At regular intervals, they will take a snapshot of their annotation database and we will use this to update the gene set, employing the Ensembl-HAVANA merge process described above. Snapshots are currently provided every 3 months for mouse, every 6 months for human, rat and zebrafish, and less frequently for pig.

### Patch annotation

**Figure 7.**
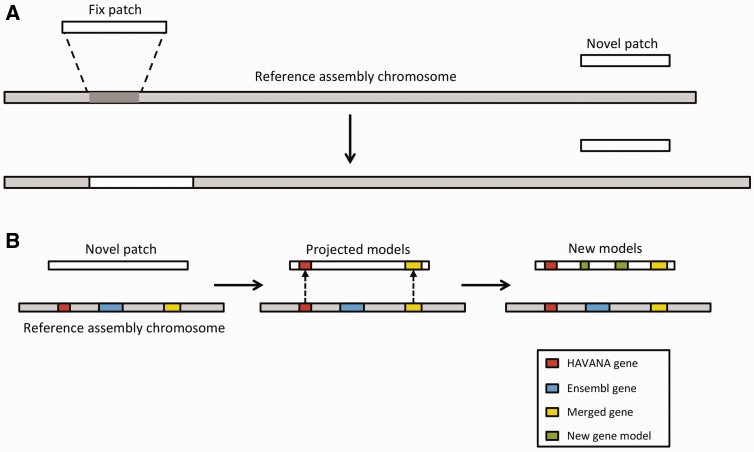
Annotation of patches. (**A**) Currently, we have two different types of patches: fix patches and novel patches. Both types are anchored to the assembly by shared sequence. Fix patches become part of the next major version of the assembly while novel patches remain as alternative sequence. (**B**) When annotating a novel patch, we first project gene models from the reference assembly. In this example, the HAVANA (red) and merged (yellow) genes are copied to the patch sequence. The Ensembl gene (blue) is not copied because the underlying genomic DNA is too different between the chromosome and the patch to enable the projection process. After projection, a patch will be annotated fully using the Ensembl annotation pipeline. In this case, two new gene models (green) have been annotated on the novel patch.

The Genome Reference Consortium (GRC) (105) currently maintains the human, mouse and zebrafish reference assemblies. Between major assembly releases, updates and improvements are released by GRC in the form of assembly patches. These patches are provided as alternate scaffolds to the primary assembly and have the purpose of either correcting known assembly errors (fix patches) or adding novel genomic sequence (novel patches) (Figure 7).

We incorporate these alternate genomic sequences, provide basic annotation on them and import all annotation on assembly patches from HAVANA. Assembly patches are anchored to the primary assembly and therefore include sequence that is identical or highly similar to the primary assembly. Our first step in providing annotation on the assembly patches is to align the patches to the primary assembly. Following the alignment, we ‘project’ annotations from the primary assembly onto the corresponding assembly patches in regions where there is high genomic similarity (Figure 7). To fill in gaps, we also use a modified version of our model-building method to add novel isoforms. This includes alignments produced by the cdna2genome step of the Targeted stage and the Similarity step. The alignments are then filtered using the TranscriptConsensus and LayerAnnotation modules, resulting in a set of new annotations on the patch region.

### RNA-seq update pipeline

We developed a method for updating an existing gene set when new RNA-seq data become available. This method is particularly relevant for species that had very little same-species data available when they were annotated, such as primates that were initially annotated using mainly human data like orang-utan. Other species with little same-species data annotated in this way include those that are distantly related to other mammals, such as platypus and opossum. The RNA-seq update method allows us to add genes and UTRs, and to lengthen truncated genes. It also identifies and removes transcript models when their splicing structure is not well supported by RNA-seq data.

This process involves first running the RNA-seq pipeline across the genome to produce a set of RNA-seq-based models. Protein-coding models from both the previous Ensembl gene set and the RNA-seq pipeline are then passed through the TranscriptConsensus, LayerAnnotation and GeneBuilder modules in order to create an updated protein-coding gene set. Pseudogenes and nonprotein-coding genes from the previous Ensembl annotation are added to finalize the gene set.

## Conclusions

We use the Ensembl gene annotation system to produce annotations for selected vertebrate genomes. During the time in which we have been producing gene sets for a wide range of vertebrate species, advances in the understanding of genome biology and new data types have presented themselves. We have embraced these developments in science and sequencing techniques to extend and improve our annotation methods, while maintaining our goal of high quality gene annotation.

We have described our more stringent choices in input data for protein and cDNA sequences, new methods for aligning these sequences to the genome and new methods for filtering the resulting alignments.

While our previously published annotation system (48) was designed to annotate only protein-coding genes, we now have systems for the annotation of short and long noncoding RNAs, immunoglobulin genes and selenoproteins. The inclusion of the HAVANA gene sets has greatly improved the annotations that we provide for human, mouse, rat, pig and zebrafish.

Furthermore, we have kept abreast of changing input data. Additional new methods in our repertoire include one for predicting transcript models from RNA-seq data, and another for updating existing gene sets using models produced by the RNA-seq pipeline. We also have a projection pipeline for annotating fragmented or higher primate genome assemblies. As sequencing technologies and software improve and mature, we will continue to improve our choice of input data and our pipelines.

With the decreasing cost in DNA sequencing, projects such as Genome10K (106) are producing a large number of genome assemblies. We are now considering how we might annotate such a large number of new genome assemblies to include in Ensembl.

## Availability

All Ensembl data and source code are freely available. Each Ensembl release is made available at http://www.ensembl.org and then maintained as an archive web site for at least 2 years after the date of initial release (see http://www.ensembl.org/info/website/archives/index.html). Ensembl is updated approximately every 3 months with new data. Not every species has sufficient new data to warrant an update for each release. The current release number and month of release are shown at the bottom of every Ensembl web page. Additionally, the data can be queried through a programmatic interface (REST or Perl API) and through the web-based Ensembl Biomart.

The full codebase for our Ensembl Gene Annotation system is available on GitHub (https://github.com/Ensembl) under an Apache 2.0 licence, and we welcome others who would like to use it. The system has been developed for our own use, as our overriding goal is to produce genome annotations and not a gene annotation program. We offer workshops and *in* *situ* training to guide collaborators in the use of the pipelines, which require a thorough understanding in order to run successfully. We continue to improve the accessibility of our annotation system and to streamline the set-up, customization and related user documentation.

## Supplementary data

Supplementary data are available at *Database* Online.
